# Discovery of Novel Derivatives of Catechin Gallate with Antimycobacterial Activity from *Kirkia wilmsii* Engl. Extracts

**DOI:** 10.3390/antibiotics15020141

**Published:** 2026-02-01

**Authors:** Nenekazi Masikantsi, Rendani Mbau, Nuhu Tukur, Peter Masoko, Gabriel Mashabela

**Affiliations:** 1 SAMRC Centre for Tuberculosis Research, Division of Molecular Biology and Human Genetics, Stellenbosch University, Francie van Zijl Drive, Tygerberg, Cape Town 7505, South Africa; mnene@sun.ac.za (N.M.); rendanim@sun.ac.za (R.M.); nuhutukur@sun.ac.za (N.T.); 2 Department of Biochemistry, Microbiology and Biotechnology, Faculty of Science and Agriculture, University of Limpopo, Private Bag X1106, Sovenga 0727, South Africa; peter.masoko@ul.ac.za

**Keywords:** *Kirkia wilmsii*, epicatechin, catechin 3′-digallate, catechin 4′-digallate, catechin 3′-monogallate, catechin 4′-monogallate, antimycobacterial, acyl migration, galloyl migration

## Abstract

*Background/Objectives:* The increase in incidences of multidrug resistance exacerbates tuberculosis-related global health challenges and underscores a call for more efforts for development of new antitubercular drugs, including the use of medicinal plants, especially those that have been used for generations by traditional healers. Despite reports of antimicrobial activity and chemical profiling of *Kirkia wilmsii* (*K. wilmsii*) extracts, chemical structures of the bioactive agents have not been elucidated. Here, we used a combination of bioactivity-guided fractionation, mass spectrometry, and nuclear magnetic resonance to purify and elucidate the chemical structure of antimycobacterial agents contained in leaf and twig extracts for *K. wilmsii*. *Results:* After overnight extraction with acetone and 90 g of dry twigs and leaves produced 5.38 g (6%) and 4.56 g (5%) of product, which displayed moderate antimycobacterial activity of 0.5 and 1 mg/mL, respectively. The antimycobacterial activity was increased six- and three-fold, respectively, after the crude extracts were subjected to solvent–solvent partitioning. Due to many bioactive fractions being obtained after silica gel chromatography purification, fraction 5 of twig extract was prioritized for further purification due to its low minimum inhibitory concentration (MIC) (0.25 mg/mL) and cytotoxicity (20%, in THP-1 cells). Sequential purification of the fraction 5 (twig extract) extracts through the C18 cartridge and high-performance liquid chromatography (HPLC) produced four fractions, which were subjected to structural elucidation. The high-resolution mass spectrometric analyses revealed that the first two eluting peaks had the same mass ion of 441.0822 *m*/*z* (M − H^−^), which corresponded to catechin monogallate, and so were the last two eluting peaks, which had a mass ion of 539.0932 *m*/*z* (M − H^−^), corresponding to catechin digallate. Further analyses by ^1^H, ^13^C, and 2D NMR confirmed the chemical structures of compounds eluting in the first two peaks on HPLC as structural isomers of catechin 3′-monogallate and catechin 4′-monogallate (MIC not determined). Similarly, compounds eluting in the last two peaks were identified as structural isomers catechin 3′-digallate and catechin 4′-digallate, with an MIC of 250 µg/mL against *Mycobacterium smegmatis* and *Mycobacterium tuberculosis* H37Rv and an MBC of 500 μg/mL against *M. smegmatis*. *Conclusions:* To the best of our knowledge, this study is the first to report the structure of catechin 3′- and 4′-digallate, their antimycobacterial activity, and the existence of acyl migration involving galloyl 3′ and 4′-hydroxyl groups of catechin ring B.

## 1. Introduction

Tuberculosis (TB), a lung disease caused by the species Mycobacterium tuberculosis complex, is one of the biggest health care challenges humanity has been battling with for centuries. Despite being a curable disease, TB still infects a lot of people, and in 2022 it was responsible for more than 1.3 million deaths worldwide [1]. Complicating TB treatment is the drug resistance, a phenomenon whereby the infecting pathogens survive otherwise lethal doses of anti-infective agents, which significantly increases the treatment failure rate as patients are treated with less effective and more toxic drugs for a long period of time [2]. To increase the number of active antitubercular arsenals, intensive research is needed to screen a variety of chemical libraries, including those derived from natural products [3]. The World Health Organization (WHO) has called for research to demonstrate the efficacy of traditional medicine due to its long historical usage in treating common symptoms of current communicable and non-communicable diseases, and medicinal plants as a viable resource for the discovery of bioactive agents [4]. It is estimated that more than 90% of the population in developing countries relies on traditional medicine, and almost 20% in South Africa [5].

*Kirkia wilmsii* Engl. (Sotho name, *Legaba* or *Modumela*), a species of the Kirkiaceae family, is one of many medicinal plants used in South Africa [6]. It is a medium to large deciduous tree with a round crown and beautiful autumn colors from April to May that is endemic to South Africa [7]. The plant is widely distributed in four northern provinces of South Africa: North West, Gauteng, Mpumalanga, and Limpopo, where it thrives on granite and dolomitic soils in dry bushveld areas or on rocky slopes [6]. Concoctions of *K. wilmsii* are widely used by Bapedi traditional healers for treatment of hypertension [8]. Additionally, the plant is used as herbal medicine for the treatment of various ailments, such as arthritis, asthma, diabetes mellitus, diarrhea, malaria, nasal congestion, and ringworm [9,10]. Antimicrobial activities of *K. wilmsii* extracts have also been recently reported, whereby significant activity against the four bacterial strains was observed in a twig decoction (average MICs of 0.51 mg/mL) against some Gram-negative and -positive bacteria [11,12]. Furthermore, the acetone extracts of *K. wilmsii* leaves were also reported to have antiplasmodial and antifungal activities [10,13].

Chemical profiling of crude extracts of *K. wilmsii* leaves, tubers, and twigs indicated the presence of different classes of compounds such as caffeic acid, gallic acid, phenolics, flavonoids, ellagic acid, cardenolide saponins, steroids, phlobatannins, and cardenolide deoxy sugars [12,14,15]. In an elegant study, Chigayo et al. reported HPLC purification of aqueous extract of *Kirkia wilmsii* tuberous roots into eight components, with four displaying antimicrobial activity against Gram-negative and -positive bacteria. Although the study did not provide chemical structures of the active agents, very useful information like UV spectra and molar absorptivity was reported, which provided critical information about the compound structures [16]. However, little to no information has been reported about the activity of *K. wilmsii* extracts against Mycobacterial species, despite the plant’s reported use in traditional medicine to treat respiratory and tuberculosis-like symptoms [9]. Here, we report activity-guided fractionation of *K. wilmsii* leaves and twig extracts using *M. smegmatis*, and structural elucidation of the bioactive agents.

## 2. Results

### 2.1. Collection of Plant Materials and Extraction

About 90 g dry leaves and 90 g twigs of *K. wilmsii* were extracted with acetone, and evaporation of the solvent yielded crude products: 5.38 g from twigs and 4.56 g from the leaves, and the samples were assessed for antimicrobial activity against *M. smegmatis*. Comparatively, twig extracts displayed higher antimycobacterial activity than the leave extracts, with MIC values of 0.5 mg/mL and 1.0 mg/mL, respectively. Additionally, more material was extracted from the twigs than the leaves (Table S1).

### 2.2. Activity-Guided Purification

#### 2.2.1. Solvent Partitioning and Silica Gel Chromatography

**Figure 1:**
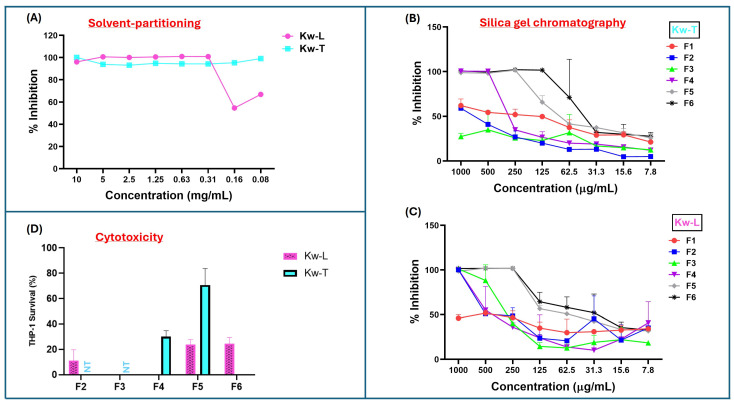
Antimicrobial and cytotoxic activities of *K. wilmsii* twig and leaf extracts at different stages of purification. Fractions were dried using speed vac and resuspended in minimal volume of 7H9 0.2% glucose medium and diluted with cell cultures to achieve about a 10–0.08 mg/mL concentration. (**A**) Antimicrobial activity against *M. smegmatis* of twig and leaf extracts after solvent-partitioning purification step. (**B**,**C**) Antimicrobial activities of fractions of twig and leaf extracts obtained after the silica gel chromatographic purification step. The values are averages of two technical repeats. (**D**) Cytotoxic effect of silica gel chromatographic fraction of twigs and leaves at 1 mg/mL concentration against THP-1 macrophages. ”NT,” which means “not tested,” indicates that the fractions were not tested, while areas without bars indicated 100% THP-1 cell killing, as indicated by dead THP-1 internalization of trypan blue dye. The values were averages of three technical repeats.

Since plant material extracted with acetone may also include compounds with intermediate polarity [17], the first step of purification was solvent partitioning using water and ethyl acetate to separate hydrophobic from hydrophilic compounds. After partitioning and removal of the solvents, 4.8 g (96%) and 3.7 g (93%) were recovered from ethyl acetate fractions of the leaves and twigs, respectively. The crude products were screened for antimycobacterial activity, and only the hydrophobic ethyl acetate fractions of both leaves and twig extracts had antimycobacterial activity. The twig fractions displayed significantly higher antimicrobial activity than leave fraction, with an MIC of ≤0.08 mg/m and 0.31 mg/mL, respectively (Figure 1A). Importantly, both the extracts had higher antimicrobial activity after solvent partitioning compared to their parent acetone crude extracts.

The bioactive hydrophobic fractions were subjected to further purification through the silica chromatography to further separate the compounds based on their affinity to the silica matrix. After the silica column was packed with petroleum ether and loaded with extracted material, the column was eluted batch-wise with 200 mL of six solvent systems made up of ethyl acetate–hexane mixtures (0%, 10%, 30%, 60% and 100%, and methanol). For both twig and leaf extract, the fractions eluted with 100% hexane (F1) contained the smallest quantity of material (Table S2), and the fractions were devoid of antimycobacterial activity (Figure 1B,C). Although fractions F2 and F3 of twig extract (Kw-T) did not have antimicrobial activity, F2 of the leaf extracts showed slight activity, with an MIC of 1 mg/mL. Antimycobacterial activity of the fractions increased slightly in fraction F3 before dropping again in F4 (Figure 1C). The most bioactive leaf fractions were F5 and F6, with both having an MIC value of 0.25 mg/mL. For the twig extract, the most active fraction was F6, with an MIC value of 0.065–0.125 mg/mL, followed by F5 and F4, with MIC values of 0.25 mg/mL and 0.5 mg/mL, respectively. Looking closely, in the elution pattern of the extracts, it was observed that the antimicrobial activity of the leaf extract was split into two, with one part eluting between F2 and F4, and the second activity eluted in F5 and F6 (Figure 1C). In contrast, the twig extract had one bioactivity eluting between F4 and F6 (Figure 1B). Notably, all of the fractions had reduced activity compared to that of their parent material before chromatographic separation, suggesting that silica gel resulted in either a loss or a spreading of antimycobacterial activity.

#### 2.2.2. Solid-Phase Extraction and HPLC Purification on C18 Matrix

Fractions that displayed the highest antimycobacterial activity while eliciting the lowest cytotoxicity against THP-1 cells were prioritized for the next step of purification (as the work was carried out in the context of drug discovery). To that end, cytotoxicity of all the fractions of twigs and leaves were evaluated. Three fractions, F2, F3, and F4 of the leaf extracts, had higher cytotoxicity, with fractions 3 and 4 almost completely killing the cells (Figure 1D). Fractions F5 and F6 were also significantly cytotoxic, as 1 mg/mL concentration killed 75% of the cells (Figure 1D). Fractions F2 and F3 of twig extract were not screened for cytotoxicity due their lower antimycobacterial activity or lack thereof (Figure 1B). Treatment with 1 mg/mL of fraction F4 killed 70% of THP-1 cells, while over 70% survived F5 at the same concentration. In contrast, fraction F6 displayed very high cytotoxicity, almost killing all the macrophages (Figure 1D). On the basis of low cytotoxicity and high antimicrobial activity, F5 of the twig extract was selected for further purification.

**Figure 2:**
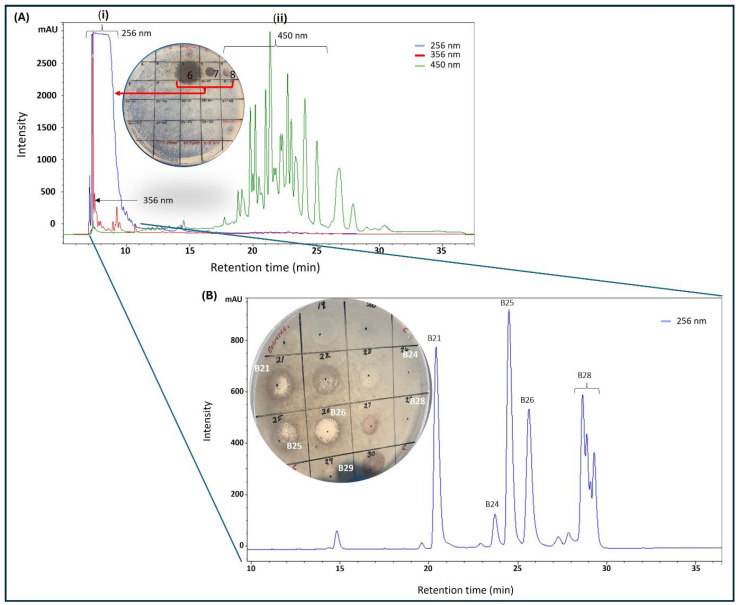
HPLC purification of silica gel (fraction 5, Figure 1B) of *K. wilmsii* twig extract. (**A**) Silica gel fraction 5 material was diluted with distilled water containing 0.1% formic acid to achieve 2.5% acetonitrile before loading onto preparative HPLC-DAD equipped with a C18 column, and the column was eluted in a 50 min gradient that achieved 90% of acetonitrile (solvent A: 0.1% formic acid; solvent B: 0.1% formic acid in acetonitrile). Fractions eluting between 6 and 10 min (i) displayed strong absorption at 265 nm and fraction eluting between 18 and 28 min (ii) displayed strong absorption at 450 nm. (**B**) HPLC chromatogram of early eluent of the first chromatography (**A**) and/or a breakthrough of the C18 cartridge preconditioned in 20% acetonitrile solution (see “Preparation of 20% Acetonitrile Breakthrough” in Section 4.3.3). Material from the breakthrough and/or early eluent was diluted with distilled water containing 0.1% formic acid solution to achieve an acetonitrile content of 2.5% before being loaded onto the preparative HPLC-DAD equipped with the C18 column. The column was eluted as described in the methods. In (**A**,**B**), the compounds were recovered by purification using a C18 cartridge, as described in the methods (see “Recovery of Compounds from HPLC Fractions” in Section 4.3.3) and antimicrobial activity was tested against *M. smegmatis*.

Purification with C18 matrix (cartridge and preparative HPLC column) required prior optimization before multiple injections were made. A small portion of twig extract F5 (10 mg), obtained from the silica chromatography, was loaded onto a C18 preparative HPLC column and eluted at 5 mL/min with a solvent gradient made up of distilled water and acetonitrile, both containing 0.1% formic acid, from 5% to 95% acetonitrile over 50 min to collect 50 × 5 mL fractions. From each fraction, displaying absorbance at either 256, 356, or 450 nm, 50 µL was aliquoted into Eppendorf tubes and solvent was removed by speed-vac at 40 °C before being reconstituted in 20 µL of 25% (*v*/*v*) ethanol and distilled water and then diluted with distilled water to make 100 µL (5% ethanol). The samples were screened for antimicrobial activity by spot assay on an agar plate containing live *M. smegmatis* cells. Much of the antimicrobial activity was concentrated in fraction 6, although slight activity was also found in fractions 7 and 8, all of which had significant absorbance at 256 nm (Figure 2A). Notably, no activity was found in fractions 18–28, which displayed significant absorbance at 450 nm (Figure 2A). The early elution of the bioactive fractions provided some clue about the structural nature of the bioactive agents, that they were significantly polar and had loose binding to the C18 matrices. With that in mind, a new purification method was formulated, in which the sample was loaded to the C18 cartridge preconditioned with 20% acetonitrile in order to recover the bioactive fraction in the breakthrough, while contaminants were retained on the column, eluted with absolute ethanol, and discarded.

The bioactive fraction was diluted with distilled water four-fold to achieve 5% acetonitrile before being reloaded onto a preparative C18 HPLC-DAD that was preconditioned with 5% acetonitrile. Gradually increasing acetonitrile concentration resolved the bioactive fraction into four significant peaks with different retention times 21 min (B21), 25 min (B25), 26 min (B26), and 28 min (B28) (Figure 2B), which were collected and processed separately. Peak purity was assessed by HPLC prior to HRMS analysis by diluting the freshly collected peak fraction with 0.1% formic acid in distilled water to achieve about 5% acetonitrile followed by injection into the preparative C18 HPLC. To our surprise, a sample collected from fraction B21 produced two peaks with equal intensities, one at its original retention time of 21 min and another at 25 min (similar to B25, as seen in Figure 2B), providing the first clue that B21 and B25 might be structural isomers undergoing isomerization in solution (Figure S1A). Similarly, reinjection of fraction B26 produced a new signal with the same retention time of 28 min, similar to B28 (Figure S1B). Subsequent assessment of antimicrobial activity by spot assay showed the B26 peak as the most active fraction, as seen on the plate in Figure 2B. Significant but lower activity was also observed on peaks B21 and B25 (plate in Figure 2B). Slight activity was observed on B28, while no activity was observed on fractions eluting at 15 and 24 min. All the purified compounds produced a strong UV absorption at 275 nm, with a weaker secondary band at 282 nm, as measured by HPLC-DAD (Figure S2).

### 2.3. Structural Elucidation

**Figure 3:**
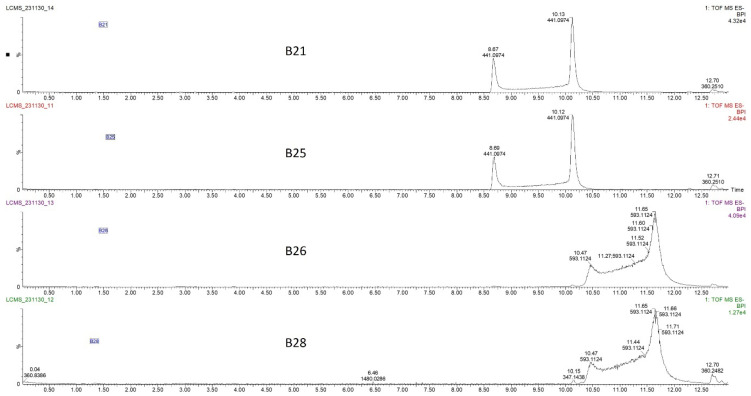
Liquid chromatography–high-resolution mass chromatogram (LC-HRMS) of compounds B21, B25, B26, and B28 collected from the C18 preparative HPLC at retention times of 21, 24, 25, and 28 min (Figure 2B). Fractions B21 and B25 had the same mass chromatogram mass ions on TOF MS ES-, 441.0974 [M − H]^−^, despite having different retention times, indicating that they were structural isomers. Similarly, fractions B26 and B28 had the same mass ion of 593.1124 [M − H]^−^ on TOF MS ES chromatograms but different retention times.

**Figure 4:**
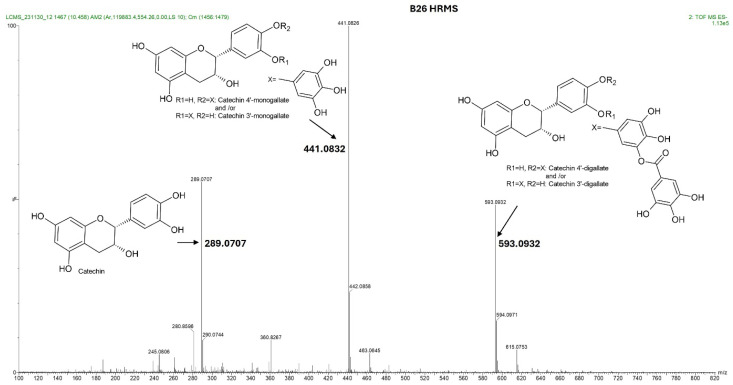
Total ion chromatogram showing the fragmentation pattern acquired by IMS QTOF ESI in the negative mode of the compound contained in fraction B26 from *K. wilmsii* twig acetone extract. Compound B26 was obtained from a preparative HPLC-DAD equipped with the C18 column and eluted at a retention time of 26 min (shown in Figure 2B). The total ion chromatogram of compound B26 showed three fragment ions, two of which differed, with the larger fragment having the same mass, 152, which corresponded to two galloyl moiety, and one with a molecular weight matching that of epicatechin/catechin. Complete structural analyses of compound B26 using 1D and 2D NMR and UV spectra confirmed that the galloyl groups were continuously moving between C-3′ and C-4′ of the epicatechin/catechin ring B.

The four HPLC fractions (B21, B25, B26, and B28) were further analyzed by high-resolution mass spectrometry coupled to analytical C18 HPLC (LC-HRMS). Despite having different retention times, compounds B21 and B25 had the same ESI-QTOF MS (*m*/*z*: 441.0822, [M − H^−^]), corresponding to the molecular formula C_22_H_17_O_10_ (Figure 3 and Figure S3). Similarly, compounds B26 and B28 had the same ESI-QTOF MS (*m*/*z*: 593.0937, [M − H^−^]) and a molecular formula of C_29_H_21_O_14_ (Figure 3 and Figure S3). Importantly, fragmentation of compound B26 produced two dominant ions (*m*/*z*: 441.0826, [M − H^−^] and *m*/*z*: 289.0707, [M − H^−^]) (Figure 4). Since B21 and B25 are the same size as fragment B26 (441.0822 and 441.0826, respectively), we deduced that compounds B21 and B25 were products of compound B26 after the loss of a moiety with a molecular weight of about *m*/*z*: 152.0101. Interestingly, it was noticed that the two fragments of compound B26 (289.0707 and 441.0832) had the same mass difference (Figure 4), suggesting that compound B26 consisted of two molecules of the same molecular weight (152 g/mol). Comparatively, the UV absorption spectrum of B26 was found to be similar to (±)-epicatechin/(±)-catechin gallates [18,19]. Since catechins have a molecular weight of 290 g/mol, which is similar to the smaller molecular ion of B26 (*m*/*z*: 289.0707, [M − H]^−^), and a galloyl group has a molecular weight of 153 g/mol, we concluded that compounds B21/B25 and compounds B26/B28 were epicatechin/catechin derivatives containing a mono- and a digallate group, respectively. The compounds were then just referred to as catechins, as no particular reference to a specific isomer could be made.

**Figure 5:**
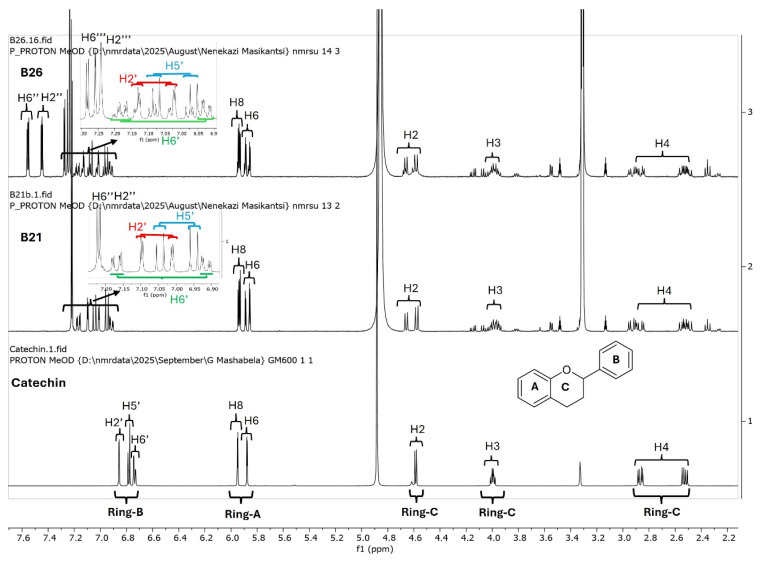
Overlays of ^1^H NMR spectra of catechin (commercial), compound B21 and compound B26. Compounds B21 and B26, together with the respective structural isomers B25 and B28, were purified from *K. wilmsii* silica gel extracts by C18 HPLC. The spectra showed an increasing up-field shift in ring B protons in compounds B21 and B26 compared to catechin. Also worth noting in B21 and B26 is a pair of doublets belonging to the ring B protons, indicating the presence of an equivalent mixture of structural isomers.

**Figure 6:**
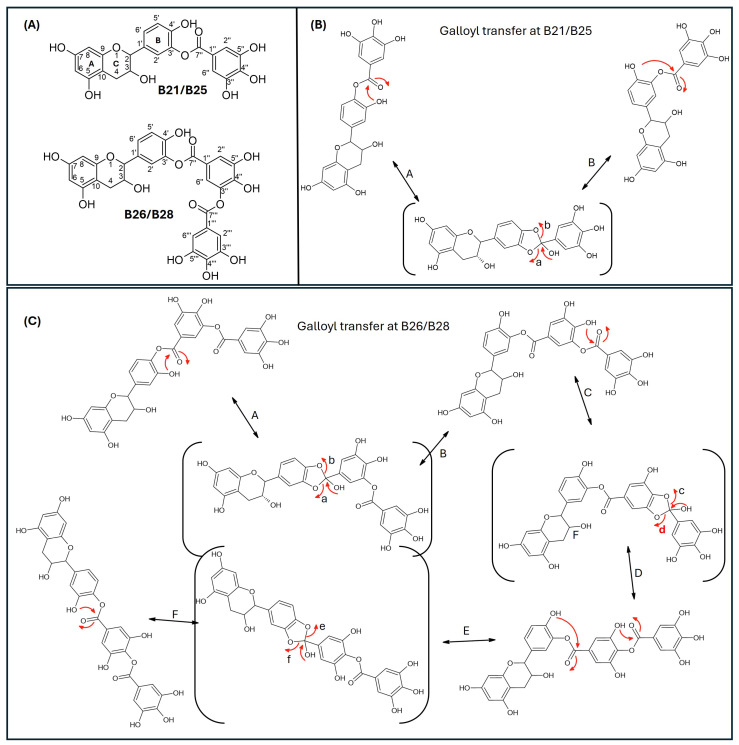
Proposed mechanism of galloyl migration in compounds B21/B25 and B26/B28 involving C-3′ and C-4′ hydroxyls on ring B of the epicatechin/catechin primary structure. (**A**) Compounds B21/B25 and B66/B28 are structural isomer pairs purified from twig acetone extracts of *K. wilmsii*. (**B**) Movement of the galloyl group between C-3’ and C-4’ hydroxyl on ring-B of the epicatechin/catechin primary structure leading to the formation of structural isomers, B21/B25. (**C**) Movement of the digalloyl group between C-3’ and C-4’ hydroxyl on ring-B of the epicatechin/catechin primary structure leading to the formation of structural isomers, B26/B26. The red arrows indicate electron movement, double edge arrows denote reversibility of the reactions, chemical structures in the parenthesis denote reaction transition states, and the lowercase letters at the head of the red arrows and the uppercase letters besides double edge arrows denote reaction paths.

**Figure 7:**
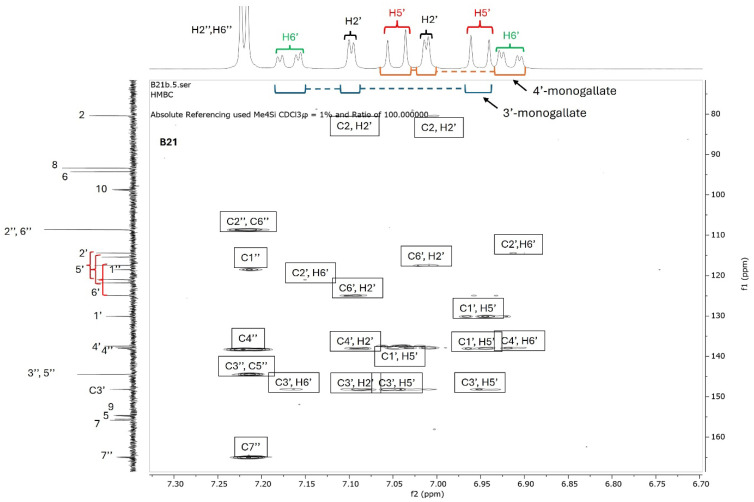
HMBC spectrum of compound B21. Compound B21 was purified from twig acetone extract of *K. wimsii* as a structural isomer of B25, which eluted at different times on C18 HPLC. The spectrum showed de-shielding of carbons and protons on ring A from the structural isomers produced during galloyl migration occurring between C-3′ and C-4′ hydroxyls. For example, the shielded H-6′ doublet of the doublets of epicatechin/catechin 4′-monogallate at δ 6.90–6.95 were highly de-shielded to δ 7.5–7.2 when the galloyl group moved to the C-3′ position on ring B. Similar proton de-shielding was also observed for the H-2′ and H-5′ doublets. The de-shielding effect was also noted on the carbon spectrum, as marked by red bracket parentheses.

Since catechins have five hydroxyl groups (two on ring A, one on ring C and two on ring B), the next challenge was to unravel the galloyl group positions of our pure compounds on the catechin structures using NMR spectroscopy. This was possible because the presence of a galloyl group would decrease the electron density and push nuclei up-field. Analyses of individual and stacked ^1^H and ^13^C spectra of compounds B21 and B26, in comparison to those of commercial catechin, showed an up-field spectral shift of ring B protons on B21 and B26 spectra, confirming the presence of an electron withdrawing group like a galloyl group exclusively on ring B (Figure 5 and Figures S4–S8). With ring B having two hydroxyl groups, it could easily be assumed that compounds B26/B28 were catechin digallates with one galloyl group on C-3′ and C-4′, respectively, while compounds B21/B25 were catechin monogallates with a galloyl group at either C-3′ or C-4′. However, further analysis of B26 ^1^H NMR showed two highly de-shielded proton peaks, at δ 7.48 ppm and 7.58 ppm, significantly up-field of the gallate proton peaks at δ 7.20–7.25 ppm (Figure 5 and Figure S5), indicating that the two galloyl groups were bonded to each other, whereby the attachment of the second galloyl group in the *meta-* position of the first galloyl would de-shield the two protons on the first galloyl ring (H-2″ and H-6″) unequally, with H-6″ being de-shielded the most, as it would be in the *para*- position of the second galloyl group, as previously reported in digallic acid [20,21,22]. Compound B26’s ^1^H NMR spectrum also showed another doublet very close to the gallate protons at δ 7.25–7.30 ppm, indicative of an alternative linkage of the second galloyl group through the *para*- position to the first galloyl group (Figure 5 and Figure S5). With that insight, we then reasoned that the occurrence of both *para*- and *meta*-linked digallate would produce structural isomers with different retention times on C18 HPLC, the phenomenon we initially observed (Figure S1A,B and Figure 3) but failed to explain without the NMR information. A similar phenomenon called acyl migration was previously observed in digallic acid molecules, whereby an activated oxygen on the *para*-hydroxyl group attacked the carbonyl at the *meta*- position and vice-versa, resulting in acyl migration producing equivalent mixtures with different retention times on C18 HPLC [20,21,23]. Therefore, we concluded that the equivalent mixtures of compound B21 and B25 arose from galloyl migration in solution between C-3′ and C-4′ hydroxyls on catechin ring B (Figure S4 and Figure 6A,B). Indeed, analyses of B21 ^13^C NMR clearly confirmed the presence of six carbons at δ 115–125 ppm, where C-2′, C-5′, and C-6′ of ring B are located, indicating a carbon shifting as the galloyl group migrated between C-3′ and C-4′ on ring B (Figures S6 and S8). More evidence supporting the occurrence of 3′–4′ acyl migration in our study was provided in the detailed HMBC spectrum of B21 (Figure 7). For example, when the galloyl group was in the C-3′ position, the H-2′ and H-6′ protons were de-shielded, as they were in the *ortho*- and *para*- positions to the galloyl group, respectively, while the C-4′ galloyl’s position resulted in H-5′ proton de-shielding (Figure 7). Evidence for the galloyl migration was also observed in B26/B28 (Figure 5, Figures S5 and S7–S10), although it was more complex. HMBC of commercial catechin was used to accurately assign protons and carbons in the primary catechin structures in the purified compounds (Figure S11). We proposed that galloyl migration in compound B26/B28 was more complex, as it occurred on both catechin ring B and the first galloyl group (Figure 6C and Figure S9).

In conclusion, compounds B26/B28, identified as the novel structural isomers of catechin 3′-digallate and catechin 4′-digallete, were obtained as a pale yellow amorphous powder, DAD Ab(max) (272–278 nm, with a shoulder at around 290 nm), LC/MS ESI^−^: *m/z* 593.0937 [M − H]^−^; calc. for [C_29_H_21_O_14_]^−^: 593.0937. ^1^H NMR (400 MHz, Methanol-d4) δ 7.56 (dd, J = 3.4, 2.1 Hz, 1H), 7.45 (dd, J = 2.9, 2.1 Hz, 1H), 7.30–7.23 (m, 2H), 7.23–7.11 (m, 1H), 7.11–7.00 (m, 1H), 7.00–6.89 (m, 1H), 5.94 (dt, J = 4.5, 2.5 Hz, 1H), 5.92–5.83 (m, 1H), 4.67 (dd, J = 7.4, 4.6 Hz, 1H), 4.63–4.55 (m, 1H), 4.04–3.94 (m, 1H), 2.90 (ddd, J = 25.5, 16.1, 5.5 Hz, 1H), 2.52 (ddd, J = 16.1, 12.4, 8.5 Hz, 1H). ^13^C NMR (101 MHz, MeOD) δ 166.63, 166.39, 166.27, 157.93, 157.88, 157.66, 157.59, 156.90, 156.71, 151.93, 150.27, 147.74, 146.63, 146.55, 145.05, 140.53, 140.26, 140.04, 139.91, 139.73, 132.30, 127.18, 123.90, 123.14, 120.69, 120.65, 120.46, 119.62, 118.10, 117.65, 116.64, 115.35, 110.96, 110.82, 110.42, 110.03, 100.96, 100.75, 96.43, 95.52, 82.57, 82.52, 69.02, 29.09, 28.44. Compounds B21/B25, identified as structural isomers of catechin 3′-monogallate and catechin 4′-monogallete, were obtained as a pale yellow amorphous powder, DAD Ab(max) (272–278 nm, with a shoulder at around 290 nm), LC/MS ESI^−^: *m/z* 441.0822 [M − H]^−^; calc. for [C_22_H_17_O_10_]^−^: 441.0827.

### 2.4. Antimycobacterial Activities of Purified Agent

The low antimycobacterial activity of B21 and B25 compared to that of B26 implicated the number of gallate groups as contributors to the higher antimycobacterial activity of compound B26 than compound B21 and B25, as observed in Figure 2B. To test this hypothesis, we assayed gallic acid and tannic acid (a compound with contain gallate groups) for antimycobacterial activity alongside catechin and B26. Gallic acid had the highest activity, with an MIC of 62.5 μg/mL, followed by tannic acid at 125 μg/mL, B26 at 250 μg/mL, and catechin at 500 μg/mL on *M. smegmatis*, thereby providing strong scientific evidence implicating the gallate group in increasing the antimycobacterial activity of flavonoid (Figure S12A). Interestingly, the minimum bactericidal activity (MBC) was found to be 500 μg/mL (Figure S12B), which was just one order of magnitude more than that of the MIC. Screening of compound B26 against *M. tuberculosis* produced an MIC of 250 μg/mL, similar to *M. smegmatis*, although gallic acid and tannic acid were less active against *M. tuberculosis* than *M. smegmatis* (Table S3).

## 3. Discussion and Conclusions

The use of *K. wilmsii* in many concoctions used for treatment of tuberculosis has been reported by some Bapedi traditional healers [9], making the plant an ideal candidate for the discovery of new antitubercular agents. Despite very limited studies reporting the detection of some biologically active compounds, no report on the purification of active agents from *Kirkia* species has been found. Nevertheless, notable to good antimicrobial activity of *K. wilmsii* extracts against some human bacterial, plasmodial, and fungal pathogens, such as *E. coli* and falciparum, have been reported. Here, we reported notable antimicrobial activity of *K. wilmsii* acetone extract. Subsequent activity-guided fractionation of the twig extract led to the purification and identification of a mixture of catechin isomers, catechin 3′-digallate and catechin 4′-digallate, as the most active antimycobacterial agents. The catechin derivatives displayed moderate antimycobacterial activity, 250 μg/mL, which compared well with the previously reported catechin MIC range of 0.125–2 mg/mL in Gram-positive and Gram-negative bacteria [24,25]. Significantly, the final compound, B26, was less active than the earlier impure fraction, suggesting a loss of activity of synergistic partners during purification. As antimicrobial agents, catechins were found to potentiate antimicrobial activities of many cell wall-targeting antibiotics, presumably through perforation of the bacterial cell membrane, altering its fluidity and damaging its integrity, which ultimately destabilizes the cell [26]. Since galloylation at positions 3, 5, and 7 renders catechin more biologically superior than those lacking the galloyl group, it is critical that the newly discovered catechin gallates be fully characterized [27,28]. Furthermore, our study also provided detailed evidence confirming, for the first time, the occurrence of galloyl migration involving C-3′ and C-4′ hydroxyls of the catechin ring B, resulting in structural isomers with different retention time on C18 matrix. Although this phenomenon is well characterized in carbohydrates and gallic acid, our study was the first to report galloyl migration in catechins [20,29]. This dynamic in-solution catechin isomerization could be the reason behind the failure to purify catechin 3′- and 4′-monogallate and other digalloyl derivatives in their natural forms, leading the researchers to derivatization prior to purification [21,30,31]. Since then, no report has been made of a successful purification of catechin 3′- or 4′-monogallate (compounds B21 and B25), while to the best of our knowledge, the biological existence or purification of catechin 3′- or 4′-digallate (compounds B26 and B28), even in their derivatized forms, has never been reported. However, on the basis of UV spectral similarities, it is probable that B26 could be the antimicrobial agent contained in the HPLC component named peak 5, one of the four fractions and reported by Chigayo et al. to have antibacterial activity [16]. The next work in our laboratory will focus on elucidating the mechanism of action and finding the target of the newly discovered catechin derivatives, quantifying the catechins and catechin gallates in different tissues of the *K. wilmsii* plant, and investigating the prevalence the catechin derivatives in other *Kirkia* plant species. Other related work will establish whether the novel gallate derivatives possess antioxidants associated with their catechin parent, which have been implicated in alleviating diabetes and hypertension risk factors [32,33,34,35,36]. Such studies will validate the use of *K. wilmsii* containing concoctions by Bapedi traditional healers the treatment of diabetes and hypertension [8,37] and establish their relevance in other useful applications [38].

## 4. Materials and Methods

### 4.1. Growth Media and Bacterial Strain

*M. smegmatis* MC2-155 or *M. tuberculosis* H37Rv cultures were removed from an 80 °C freezer and allowed to thaw on ice before being transferred into a 25 mL culture flask containing 9 mL 7H9 (Middlebrook, Bolton, UK) medium supplemented with 10% oleic acid, albumin, dextrose, and catalase (OADC (Middlebrook)), 0.05% Tween-80. The cultures were grown at 37 °C in a shaking incubator (IncoShake, Labotec, Midrand, South Africa) overnight for *M. smegmatis* or without shaking for 7 days for *M. tuberculosis*. The pellets were harvested by centrifugation at 5000 rpm for 10 min at room temperature, and the supernatant was discarded. For *M. smegmatis*, the pellet was resuspended in 10 mL 7H9 medium containing 0.2% glucose (Sigma, St. Louis, MO, USA), 0.05% Tween-80, to make OD_600nm_ of 0.05 before being incubated overnight in a shaking incubator to obtain OD_600nm_ of 0.4–0.8. For *M. tuberculosis*, the pellet was resuspended to the same density in 7H9 medium supplemented with 10% OADC, 0.05% Tween-80. The cultures were ready for use in subsequent assays.

### 4.2. Plant Collection and Extraction

Plant material consisting of 400 g each of twigs (Kw-T) and leaves (Kw-L) of *K. wilmsii* were collected in April at Ga-Mongatane village, Limpopo province, with the identity confirmed at the University of Limpopo (specimen number: S94), and kept and processed separately. The material was dried at room temperature for a month in a well-ventilated place, away from direct sunlight. Dried material was first crushed by hand before being finely crushed and homogenized by a blending machine (RAF, Zhejiang, China). The fine plant material (30 g) was transferred into a 1 L glass beaker containing a magnetic stirrer bar (Sigma, Saint Louis, MO, USA), soaked in 300 mL acetone (HPLC grade), and covered with aluminum foil. The mixture was stirred at room temperature for 3 h in a fume hood and left to stand overnight away from direct sunlight. Subsequently, the mixture was stirred for 10 min and left to stand for 1 h. The supernatant was decanted into a clean glass beaker and 100 mL acetone was added to the slurry, stirred for 3 h, and allowed to stand for 1 h. The supernatant was removed and mixed with the earlier batch and the slurry was discarded. The extraction procedure was repeated two more times (90 g extracted). The pooled supernatants (3 × 400 mL), in a beaker, were covered with aluminum foil and allowed to stand in a fume hood overnight. The extract was slowly poured into a new beaker through a cotton cloth before being filtered through a Whatman paper (Sigma). A celite (1 g) was added to the extract and mixed. The slurry was filtered through a fitted filter containing a layer of celite that had been washed with acetone. The solvent was removed using a rotatory evaporator (BUCHI, Meierseggstrasse, CH, Switzerland) to obtain a dark brown sticky material.

### 4.3. Activity-Guided Fractionation

#### 4.3.1. Solvent Partitioning

The leaf (5 g) and twig (4 g) samples were dissolved in minimal amount of acetone before mixing with 300 mL ethyl acetate (Sigma, analytical grade). The solution was transferred into a 1 L separation glass funnel, followed by the addition of 500 mL water. The mixture was vigorously mixed by shaking for about 3 min and put on a stand to settle. The aqueous layer (bottom) was removed and put into a glass beaker, and 500 mL distilled water was added to the separation funnel. The mixture was mixed vigorously by shaking for about 3 min and allowed to stand. The aqueous layer was removed and mixed with the previous one, and the ethyl acetate fraction was collected in a beaker. The ethyl acetate layer was removed using a rotatory evaporator to obtain a brown sticky material of leaves (4.8 g, 96%) and twigs (3.7 g, 93%). About 10 mg from each sample was dissolved in DMSO (Sigma, ACS reagent, 99.9%) to make 1 mg/mL solution and used to evaluate antimicrobial activity. For the aqueous layer, only a small fraction (1 mL each) was dried using a speed vac and resuspended in growth medium (to allow for antimicrobial assessment).

#### 4.3.2. Silica Gel Purification

Silica gel 200 mesh (Sigma), 200 g, was mixed with hexane (HPLC grade) to form a slurry that was transferred to a column that was blocked with a cotton wool to trap the resin. The bioactive material, produced through the solvent–solvent partitioning above (leaves (4.7 g) and twigs (3.5 g)), was dissolved in a minimal amount of methanol (HPLC grade) and loaded onto the column. The column was eluted sequentially with 200 mL each of solvent mixture (0%, 10%, 30%, 60%, 100% of ethyl acetate in hexane) and 200 mL methanol. The solvents were removed by a rotatory evaporator to a small amount that was transferred by a glass pipette into vials, which were left open in a fume hood overnight to produce a dark green-brown sticky paste. Small amounts of dry samples were dissolved in DMSO to make 1 mg/mL solutions and used to assay for antimicrobial activity.

#### 4.3.3. Solid-Phase Extraction (C18 Cartridge)

##### Preparation of 20% Acetonitrile Breakthrough

The C18 cartridges (Sep-Pak C18 Plus Light Cartridge, 130 mg Sorbent per Cartridge, 55–105 µm, Waters, Milford, MA, USA) were prepared by running 10 mL acetonitrile (HPLC grade) through, followed by 10 mL of 20% solution of acetonitrile in distilled water. The bioactive material, produced above after silica gel chromatography, was dissolved in acetonitrile and then diluted with distilled water to make 20% acetonitrile solution. The mixtures were immediately mixed and slowly run through C18 cartridges that had been pre-conditioned as described above. The cartridges were washed with 2 mL of 20% acetonitrile–water before being slowly eluted with 5 mL acetonitrile. About 500 µL of eluent was transferred into 2 mL Eppendorf tubes (after the empty weight was recorded), and the solvent was removed by speed vac (Concentrator Plus, Eppendorf®, Hamburg, DE, Germany) The samples were dissolved in 200 µL of ethanol, dried using a speed vac, and resuspended in ethanol to make approximately 50 μg/mL solution. The samples were diluted 1 in 50 with growth medium (7H9 broth supplemented with 10% OADC) and spotted onto a layer of *M. smegmatis* that had been spread on plates of 7H10 agar supplemented with 10% OADC. The plates were incubated for three days.

##### Recovery of Compounds from HPLC Fractions

The C18 cartridges were prepared as above and followed by running them through 10 mL of 2.5% solution of acetonitrile in distilled water. Fractions from the C18 preparative HPLC-DAD were pooled and diluted with distilled water to achieve 2.5% acetonitrile and loaded onto the prepared cartridges. The cartridges were washed with 10 mL distilled water before being eluted with 600 μL absolute ethanol into Eppendorf tubes (Greiner Bio-One, Kremsmünster, AUT) (pre-weighed), and the solvents were removed by speed vac. The dry material was stored at −20 °C until required.

#### 4.3.4. HPLC

The breakthrough obtained from the solid-phase extraction above was diluted 1 in 10 with 0.1% formic acid in water to achieve about 2.5% acetonitrile, and 5 mL of this material was analyzed on preparative HPLC using a two-solvent system, solvent A (distilled water containing 0.1% formic acid) and solvent B (acetonitrile (HPLC grade) containing 0.1% formic acid). The flow rate was maintained at 5 mL/min at room temperature. The sample was separated through a preparative C18 column (Agilent Prep 100 Å C18, 21.2 × 50 mm, 5 µm, P/N 446905-702) using the following gradient of solvent A and solvent B: 0–25 min (5–25% B), 25–30 min (25–95% B), 30–40 min (95% B), and 40–45 min (95–2.5% B). Eluting fractions were detected at 256, 356, and 450 nm wavelengths to cover diverse plant-derived chemical structures (phenolic acids, flavonoids, and carotenoids), and 5 mL fractions were collected using a fraction collector. The fractions with the corresponding absorption peaks were pooled and diluted with 0.1% formic acid solution to achieve approximately 2% acetonitrile. The diluents were loaded onto a cartridge that had been preconditioned with 2% ethanol in water solution. The cartridges were washed with 5 mL of 2% ethanol solution and eluted slowly with 300 µL ethanol and dried using a speed vac to achieve an off-white, amorphous powder. The dried material was suspended in about 100 µL of growth medium and used to assess antimicrobial activity using a spot assay. Purified active fractions were analyzed by high-resolution mass spectrometry (HRMS) and NMR (^1^H and ^13^C) for structural elucidation.

### 4.4. HRMS, NMR, and Chemical Structures

Samples were analyzed on a Waters Cyclic select IMS QTOF mass spectrometer coupled (Waters, Milford, MA, USA) to a Waters UPLC equipped with HSS T3 column with the following mass spectrometer settings: ESI probe, ESI Negative, Cone Voltage 15 V. A gradient of 100% solvent A (0.1% formic acid) to 100% solvent B (acetonitrile) was run over 12 min. The NMR 1H and 13C nuclear magnetic resonance (NMR) was measured on a 600 MHz NMR spectrometer in MeOD solvent. NMR data was analyzed using MestReNova Version 12.0.4-22023. Chemical structures were drawn using ACD/ChemSketch (Freeware).2022.2.1.

### 4.5. Antimicrobial Activity Assays

#### 4.5.1. Minimum Inhibitory Concentration (MIC)

MIC was determined using a resazurin microtiter assay (REMA) as previously described [39]. Stock solutions of drugs and extracts were prepared in DMSO. Briefly, 2-fold serial dilutions of drugs or extracts were performed in a 96-well round-bottom plate using 7H9-glucose medium (*M. smegmatis*) or 7H9 OADC (*M. tuberculosis*). *M. smegmatis* or *M. tuberculosis* culture (as prepared above) was diluted accordingly to achieve an OD_600nm_ of 0.0006 and was added in an equal volume for a total volume of 100 µL per well to the serially diluted drugs or extracts. Tannic acid, gallic acid, and catechin were all purchased from Sigma. The plates were wrapped with zip-lock bags and incubated at 37 °C for 3 days without shaking (*M. smegmatis*) or 7 days (*M. tuberculosis*), after which 10 µL of Alamar Blue (resazurin) (ThermoFisher, Waltham, MA, USA) was added. After 24 h of incubation, the plates were analyzed for fluorescence using a FluorStar machine (BMG LABTECH, Offenburg, Germany) at excitation and emission wavelengths of 544 nm and 590 nm, respectively.

#### 4.5.2. Bactericidal Concentration (MBC)

Plates were prepared as described for MIC but before resazurin was added. The suspensions were transferred into Eppendorf tubes and centrifuged at 10,000 rpm for 10 min at room temperature. The supernatants were discarded, and the pellets were resuspended in 100 µL of 7H9 medium supplemented with 10% OADC before plating on a Petri dish containing 7H10 agar supplemented with 10% OADC. The plate was incubated for three days at 37 °C.

#### 4.5.3. Zone of Inhibition Assay

Drugs or extracts in organic solvents (ethanol of DMSO) were diluted 1/50 using 7H9 broth supplemented with 10% OADC, 0.05% Tween-80, and spotted (10 µL) onto a lawn of *M. smegmatis* on a Petri dish containing 7H10 agar supplemented with 10% OADC. The plate was incubated for three days.

### 4.6. Cytotoxicity Assessment

THP-1 cells (ATCC TIB-202) were cultured in RPMI-1640 (Gibco (Grand Island, NY, USA), supplied by ThermoFisher) medium supplemented with 10% fecal bovine serum (Gibco, supplied by ThermoFisher) until more than 95% viability was achieved before the addition of 100 ng/mL phorbol 12-myristate 13-acetate (PMA) (ThermoFisher) to induce differentiation. Cells were then seeded on 96-well plates at 1 × 10^5^ cell/well density, measured using a hemocytometer. The cells were exposed to *K. wilmsii* twig and leaf extracts at 1 mg/mL. Cell viability was determined by counting viable cells using the trypan blue exclusion method, and the viability percentages were calculated relative to untreated controls.

## Data Availability

The original contributions presented in this study are included in the article/Supplementary Material. Further inquiries can be directed to the corresponding author.

## Supplementary Materials

The following supporting information can be downloaded at: https://www.mdpi.com/article/10.3390/antibiotics15020141/s1, Figure S1: Chromatograms showing re-injection of HPLC fractions that were separately collected. Figure S2: Absorption spectrum of B26 fraction. Figure S3: High-resolution mass spectra (QTOF MS ES-) of compounds B21, B25, B26, and B26 purified from *Kirkia wilmsii* extract by C18 HPLC. Figure S4: ^1^H NMR spectrum of compound B21 purified by C18 HPLC from *Kirkia wilmsii* twigs. Figure S5: ^1^H NMR spectrum of compound B26 purified by C18 HPLC from *Kirkia wilmsii* twigs. Figure S6: ^13^C NMR spectrum of compound B21 purified by C18 HPLC from *Kirkia wilmsii* twigs. Figure S7: ^1^H NMR spectrum of compound B26 purified by C18 HPLC from kirkia wilmsii twigs. Figure S8: Overlay of 13C NMR spectra of catechin (commercial), compound B21 and compound B26 fraction. Figure S9: HMBC spectrum of compound B26 that was purified by C18 HPLC from Kirkia wilmsii twigs. Figure S10: COSY spectrum of compound B26 that was purified by C18 HPLC from Kirkia wilmsii twigs. Figure S11: HMBC spectrum of commercial catechin (Sigma-Aldrich). Figure S12: Antimycobacterial effect of compound B26 in comparison to catechin- and gallic acid-containing compounds. Table S1: Table showing twig and leaf acetone extraction yield and antimycobacterial activity in *M. smegmatis*. Table S2: Silica gel chromatography fractions of *K. wilmsii* twig extract and their antimycobacterial activity against *M. smegmatis*. Table S3: Antimycobacterial activity of B26 in comparison to catechin- and gallic acid-containing compounds.
