# Supplementary material for: Discovery of Novel Derivatives of Catechin Gallate with Antimycobacterial Activity from Kirkia wilmsii Engl. Extracts
Source: Antibiotics (Basel). 2026 Feb 1;15(2):141. doi: 10.3390/antibiotics15020141 (PMC12937249; doi:10.3390/antibiotics15020141)
Supplement: Supplementary file 1 [file antibiotics-15-00141-s001.zip › Figure S1.pdf]

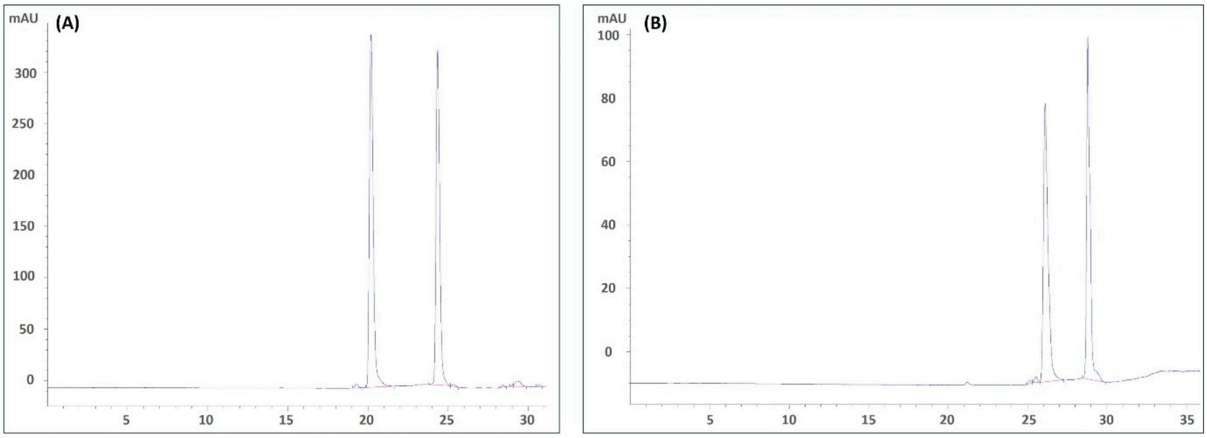

Figure S1: Chromatograms showing re-injection of HPLC fractions that has been were separately collected. In (A), fraction B21 was reinjected and it produced peaks at 25 minutes. Similarly, B26 was reinjected and it produced peaks at 26 minutes.
