# Supplementary material for: Discovery of Novel Derivatives of Catechin Gallate with Antimycobacterial Activity from Kirkia wilmsii Engl. Extracts
Source: Antibiotics (Basel). 2026 Feb 1;15(2):141. doi: 10.3390/antibiotics15020141 (PMC12937249; doi:10.3390/antibiotics15020141)
Supplement: Supplementary file 1 [file antibiotics-15-00141-s001.zip › Figure S12.pdf]

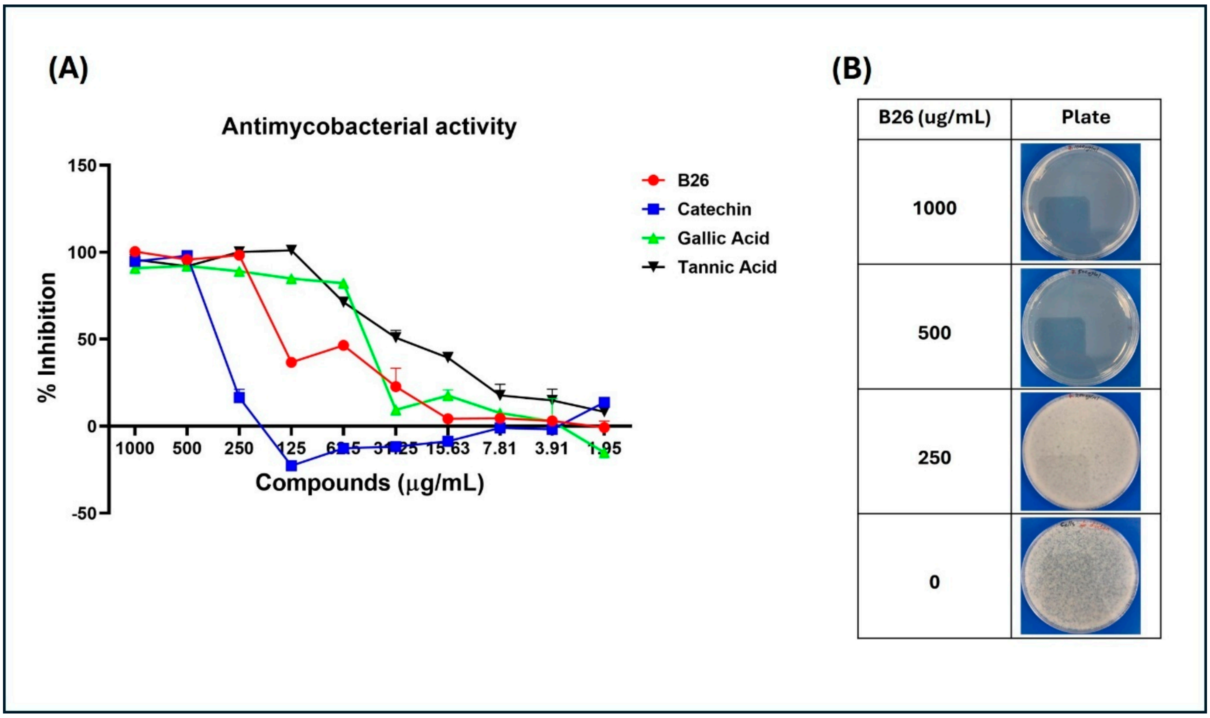


**Figure S12:** Antimycobacterial effect of compound B26 in comparison to catechin and gallic acid containing compounds. (A) Antimycobacterial activity of B26 fraction in comparison with catechin and gallate containing compounds (gallic acid and tannic acid). The compounds solubilised in DMSO were diluted in 7H9 0.2% glucose medium to afford 2% DMSO concentration before serially diluted in the same medium. The diluted compounds were aliquoted (50 uL) into 96 well plates followed by addition of 50 uL of *M. smegmatis* culture at OD of 0.0006. The plates were incubated for three days at 37°C. Resazurin was added and the fluorescence was monitored after 4 hours at excitation and emission wavelengths of 544 nm and 590-10nm. The values were averages of two technical repeats. (B) Bactericidal effect of B26 fraction. The cells were prepared as described above in A, but without resazurin addition. The wells were mixed and the suspensions were transferred into Eppendorff tubes and centrifuged at 10 000 rpm for 10 minutes at room temperature. The supernatants were discarded, and the pellets were resuspended in 100 µl of 7H9 medium supplemented with 10% OADC before plating on a petri dish containing 7H10 agar supplemented with 10% OADC. The plate was incubated for three days at 37°C
