# Supplementary material for: Discovery of Novel Derivatives of Catechin Gallate with Antimycobacterial Activity from Kirkia wilmsii Engl. Extracts
Source: Antibiotics (Basel). 2026 Feb 1;15(2):141. doi: 10.3390/antibiotics15020141 (PMC12937249; doi:10.3390/antibiotics15020141)
Supplement: Supplementary file 1 [file antibiotics-15-00141-s001.zip › Figure S2.pdf]

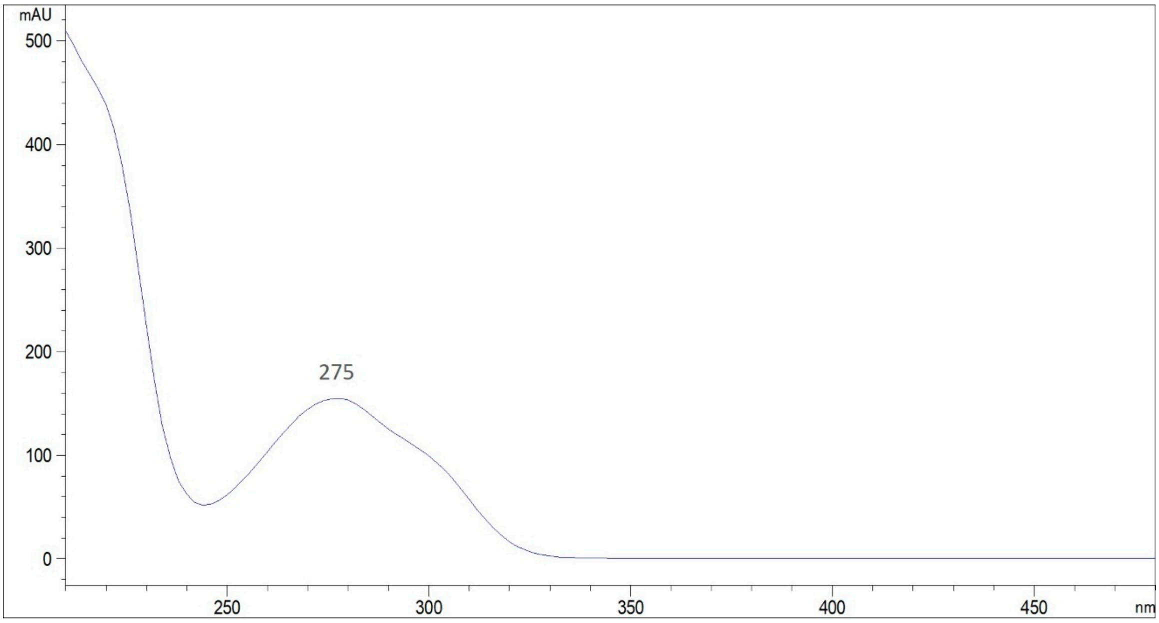

Figure S2: Absorption spectrum of B26 fraction. The compounds was obtained from *K. wilmsii* extract after purification by silica gel chromatography and C18 HPLC-DAD. The compound displayed high absorption at 275 nm and a shoulder at around 290 nm and antimycobacterial activity against *M. smegmatis*.
