# Supplementary material for: Discovery of Novel Derivatives of Catechin Gallate with Antimycobacterial Activity from Kirkia wilmsii Engl. Extracts
Source: Antibiotics (Basel). 2026 Feb 1;15(2):141. doi: 10.3390/antibiotics15020141 (PMC12937249; doi:10.3390/antibiotics15020141)
Supplement: Supplementary file 1 [file antibiotics-15-00141-s001.zip › Figure S3.pdf]

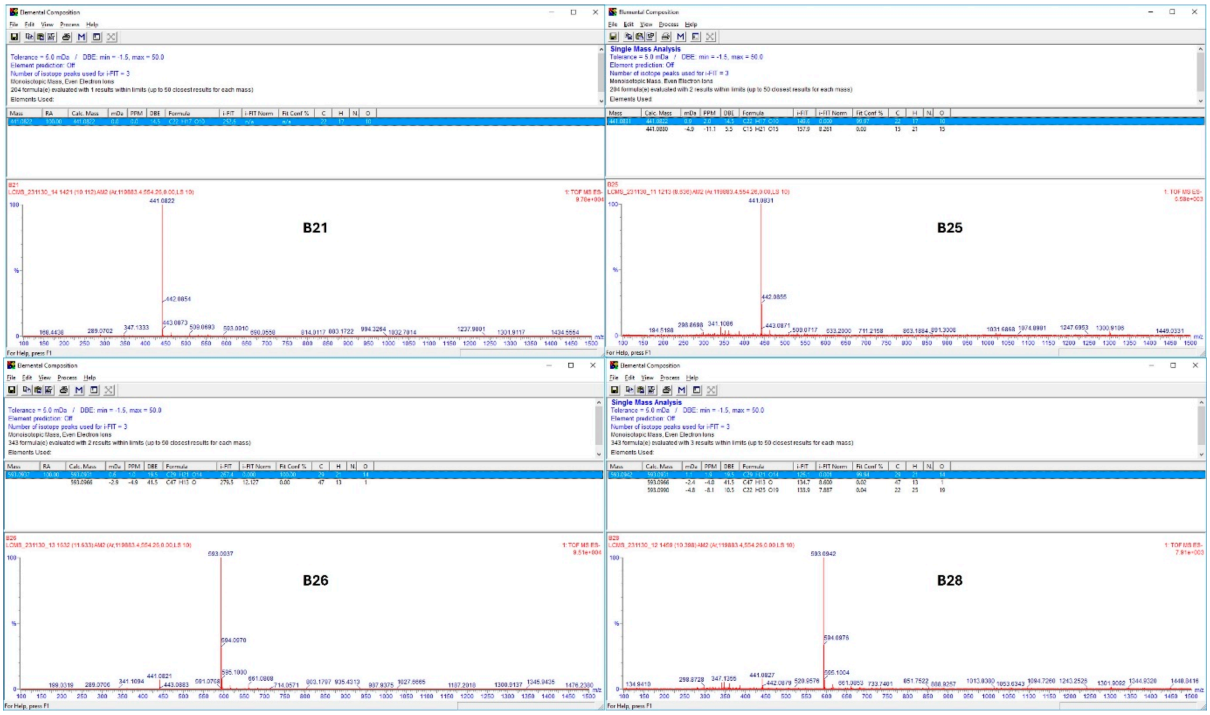

Figure S3: High resolution mass spectra (QTOF MS ES^-) of compounds B21, B25, B26 and B26 purified from *K. wilmsii* extract by C18 HPLC. Compound B21 and B25 were structural isomers with mass ion of m/z 441.0822 and 441.0821, respectively, corresponding to molecular formular $C_{22}H_{17}O_{10}$. Similarly, compounds B26 and B28 were structural isomers with mass ion of m/z 593.0937 and 593.0942, respectively, corresponding to molecular formula $C_{29}H_{21}O_{14}$.
