# Supplementary material for: Discovery of Novel Derivatives of Catechin Gallate with Antimycobacterial Activity from Kirkia wilmsii Engl. Extracts
Source: Antibiotics (Basel). 2026 Feb 1;15(2):141. doi: 10.3390/antibiotics15020141 (PMC12937249; doi:10.3390/antibiotics15020141)
Supplement: Supplementary file 1 [file antibiotics-15-00141-s001.zip › Figure S4.pdf]

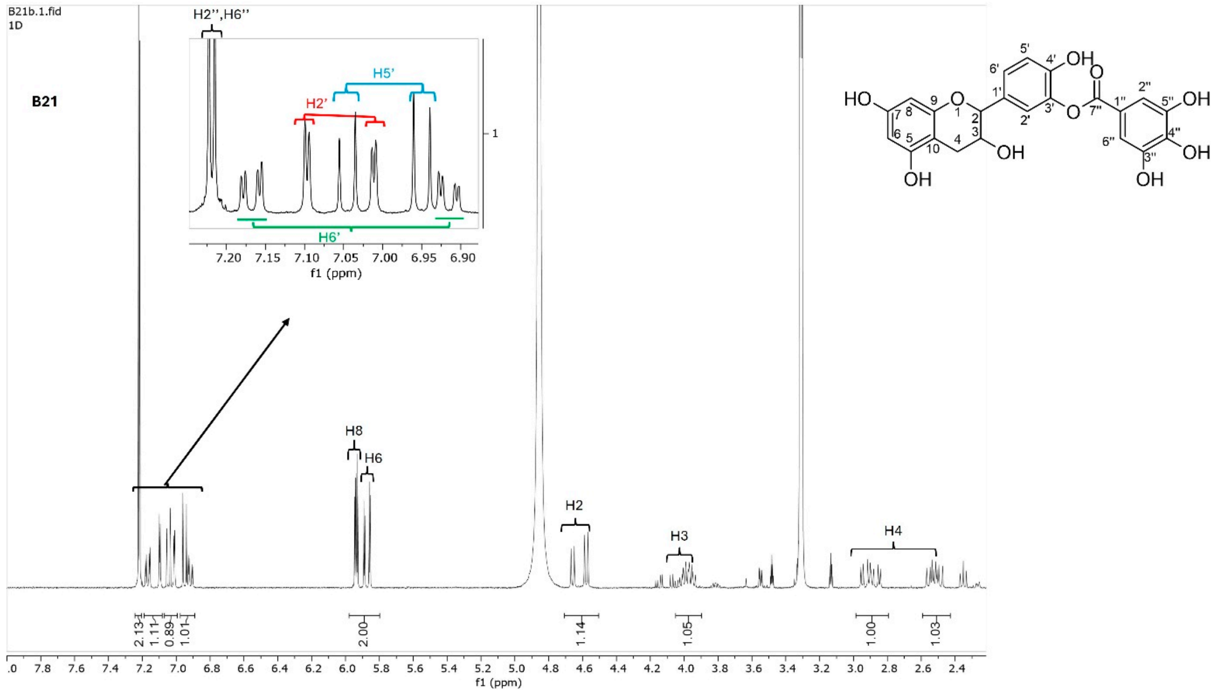

Figure S4: ^{1}H NMR spectrum of compound B21 purified by C18 HPLC from *K. wilmsii* twigs. Other than epicatechin/catechin ion signals, ring-C (H-4, H-3, H-2), ring-A (H6, H-8) and ring-B (δ 6.90 – 7.20), compound B21 showed galloyl characteristic ion signals at between δ 7.20 – 7.25. Since the two galloyl protons produce a singlet because they are equivalent, the presence of a doublet indicated the presence of an equimolar mixture. This phenomenon was also shown by the presence of pairs of doublet of doublets and doublets (insert), indicated the presents of galloyl derivatives with changes only happening at ring-B.
