# Supplementary material for: Discovery of Novel Derivatives of Catechin Gallate with Antimycobacterial Activity from Kirkia wilmsii Engl. Extracts
Source: Antibiotics (Basel). 2026 Feb 1;15(2):141. doi: 10.3390/antibiotics15020141 (PMC12937249; doi:10.3390/antibiotics15020141)
Supplement: Supplementary file 1 [file antibiotics-15-00141-s001.zip › Figure S5.pdf]

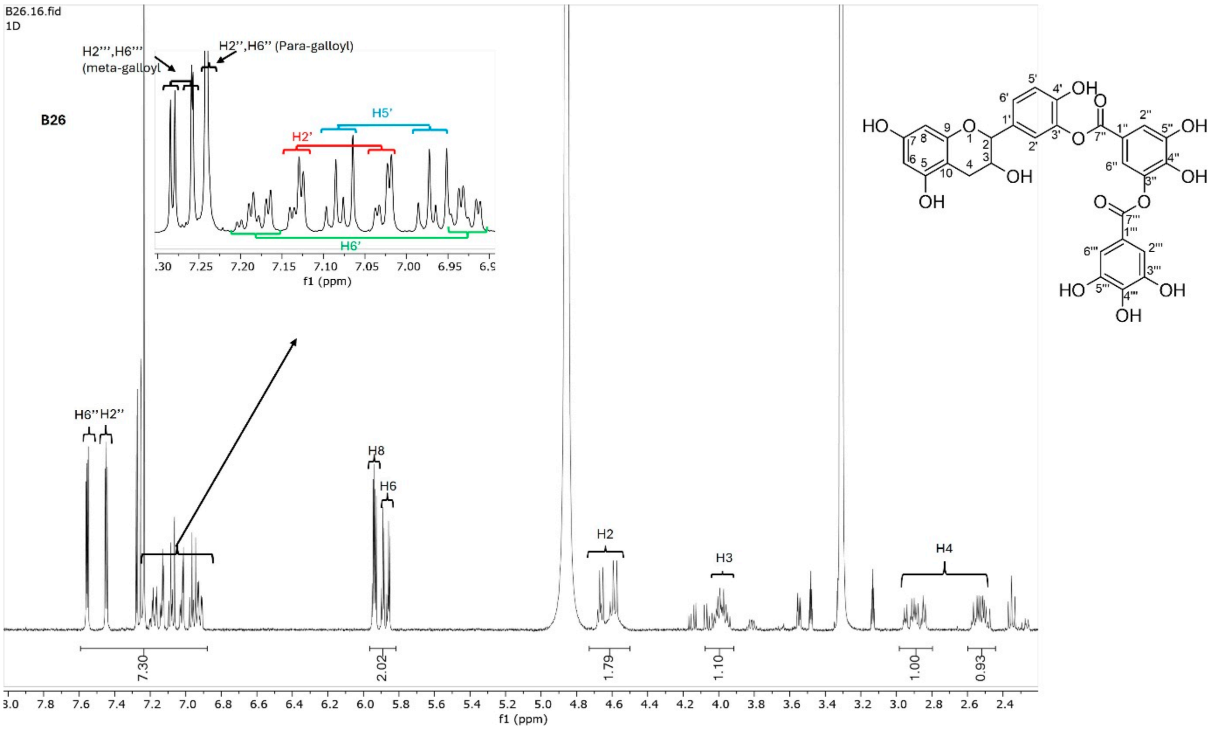

Figure S5: ^{1}H NMR spectrum of compound B26 purified by C18 HPLC from *K. wilmsii* twigs. Other than epicatechin/catechin ion signals, ring-C (H-4, H-3, H-2), ring-A (H6, H-8) and ring-B (δ 6.90 – 7.20), compound B21 showed galloyl characteristic ion signals at between δ 7.20 – 7.25. The spectrum also showed two distinct pairs of multiples, one at δ 7.25 – 7.30 and the other at δ 7.40 – 7.60 , away from intact galloyl group protons at δ 7.20 – 7.25., indicating the presence of structural isomers with galloyl groups reacting in meta- and para-positions to each other.
