# Supplementary material for: Discovery of Novel Derivatives of Catechin Gallate with Antimycobacterial Activity from Kirkia wilmsii Engl. Extracts
Source: Antibiotics (Basel). 2026 Feb 1;15(2):141. doi: 10.3390/antibiotics15020141 (PMC12937249; doi:10.3390/antibiotics15020141)
Supplement: Supplementary file 1 [file antibiotics-15-00141-s001.zip › Figure S8.pdf]

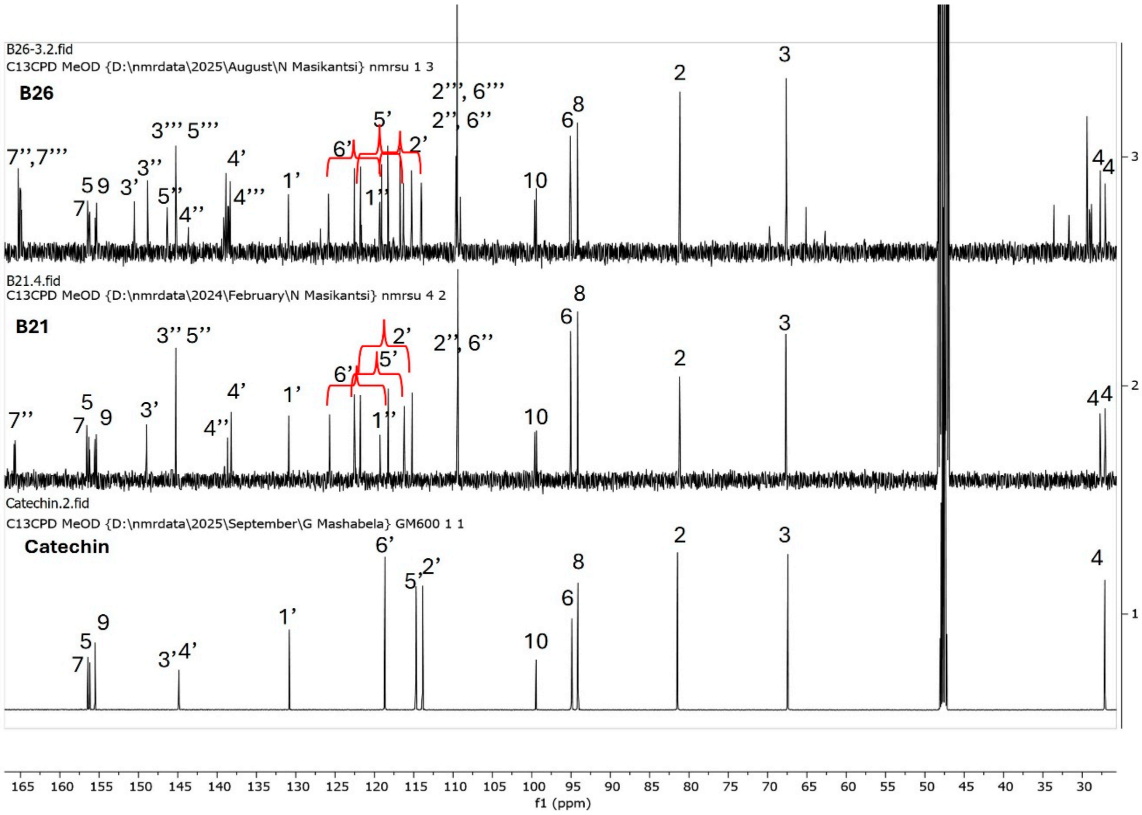

Figure S8: Overlay of ^{13}C NMR spectra of catechin (commercial), compound B21 and compound B26 fraction. Compounds B21 and B26 were purified by C18 HPLC from *K. wilmsii* twigs. There was no spectral shift across the spectra at δ 25 – 105 ppm region, suggesting that no modifications occurred in ring-A and ring-C of the parent catechin structure. The characteristic galloyl signals appeared at δ 110 ppm on compound B21, which increased in size and numbers in compound B26, indicating the presence of one galloyl group in compound B21 and two galloyl groups in compound B26.
