# Supplementary material for: Discovery of Novel Derivatives of Catechin Gallate with Antimycobacterial Activity from Kirkia wilmsii Engl. Extracts
Source: Antibiotics (Basel). 2026 Feb 1;15(2):141. doi: 10.3390/antibiotics15020141 (PMC12937249; doi:10.3390/antibiotics15020141)
Supplement: Supplementary file 1 [file antibiotics-15-00141-s001.zip › Table S1.pdf]

Table S1: Table showing twigs and leaves acetone extraction yield and antimycobacterial activity in *M. smegmatis*.

Plant part	Dry material	Product	Yield	MIC (mg/mL)
Kw-T	90 g	5.38 g	5.98%	0.5
Kw-L	90 g	4.56 g	5.1%	1.0
