# Supplementary material for: Discovery of Novel Derivatives of Catechin Gallate with Antimycobacterial Activity from Kirkia wilmsii Engl. Extracts
Source: Antibiotics (Basel). 2026 Feb 1;15(2):141. doi: 10.3390/antibiotics15020141 (PMC12937249; doi:10.3390/antibiotics15020141)
Supplement: Supplementary file 1 [file antibiotics-15-00141-s001.zip › Table S2.pdf]

Figure S2: Silica gel chromatography fractions of *K. wilmsii* twig extract and their antimycobacterial activity against *M. smegmatis*.

Solvent	Ethyl acetate percent in hexane					
	0	10	30	60	100	Methanol
Fraction	F1	F2	F3	F4	F5	F6
Mass	0.47g	0.01g	0.08g	0.87g	1.49g	1.87g
MIC (mg/mL)	>1.00	>1.00	>1.00	0.5	0.25	0.13
