# Supplementary material for: Discovery of Novel Derivatives of Catechin Gallate with Antimycobacterial Activity from Kirkia wilmsii Engl. Extracts
Source: Antibiotics (Basel). 2026 Feb 1;15(2):141. doi: 10.3390/antibiotics15020141 (PMC12937249; doi:10.3390/antibiotics15020141)
Supplement: Supplementary file 1 [file antibiotics-15-00141-s001.zip › Table S3.pdf]

Table S3: Antimycobacterial activity of B26 in comparison to catechin and gallic acid containing compounds.

	Minimum inhibitory concentration (mg/mL)	
**Compounds**	***M. smegmatis***	***M. tuberculosis***
Tannic acid	0.125	>0.250
Gallic acid	0.063	>0.250
Catechin	0.500	0.250
B26	0.250	0.250
