# Supplementary figures and images for: Discovery of Novel Derivatives of Catechin Gallate with Antimycobacterial Activity from Kirkia wilmsii Engl. Extracts
Source: Antibiotics (Basel). 2026 Feb 1;15(2):141. doi: 10.3390/antibiotics15020141 (PMC12937249; doi:10.3390/antibiotics15020141)

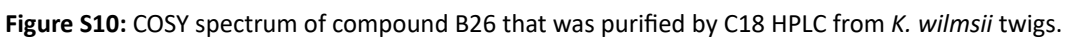

**Figure S10:** COSY spectrum of compound B26 that was purified by C18 HPLC from *K. wilmsii* twigs.

Supplement: Supplementary file 1 [file antibiotics-15-00141-s001.zip › Figure S10.pdf]

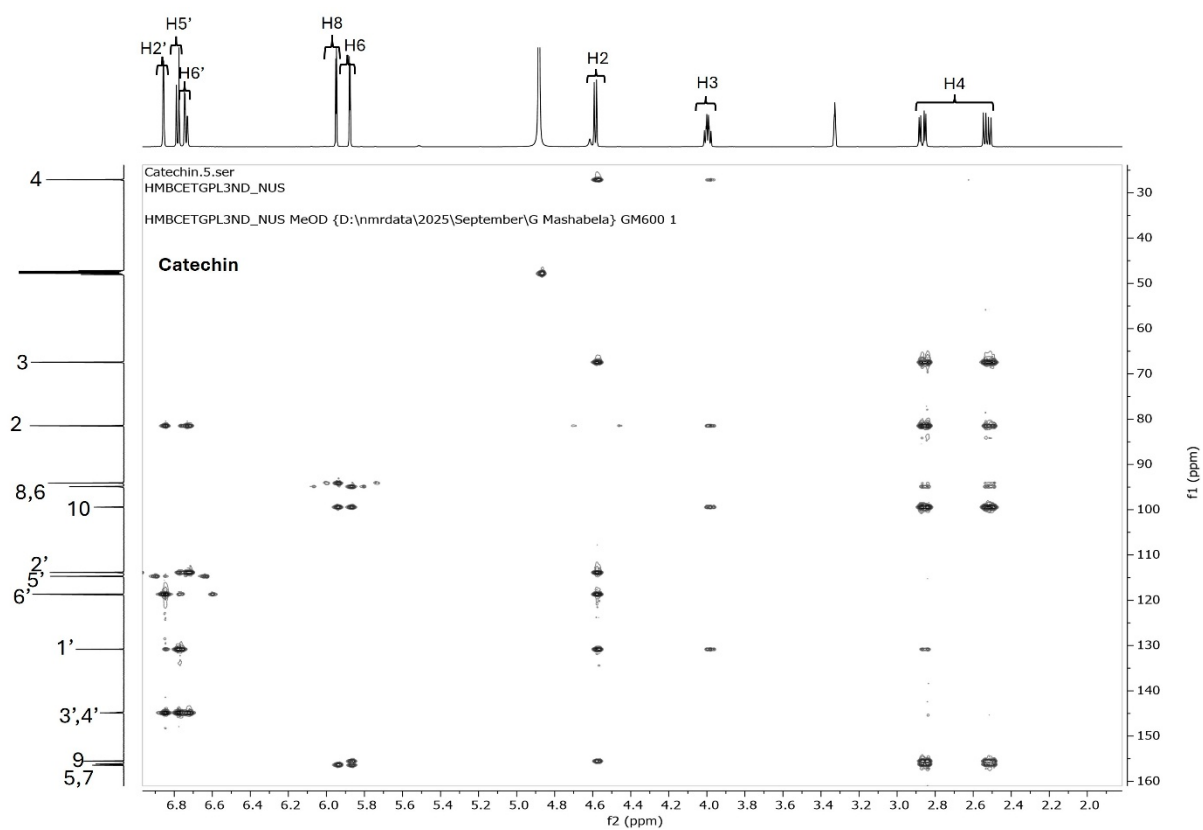

**Figure S11:** HMBC spectrum of commercial catechin (Sigma-Aldrich).

Supplement: Supplementary file 1 [file antibiotics-15-00141-s001.zip › Figure S11.pdf]

F21.4.fid  
1D

**B21**

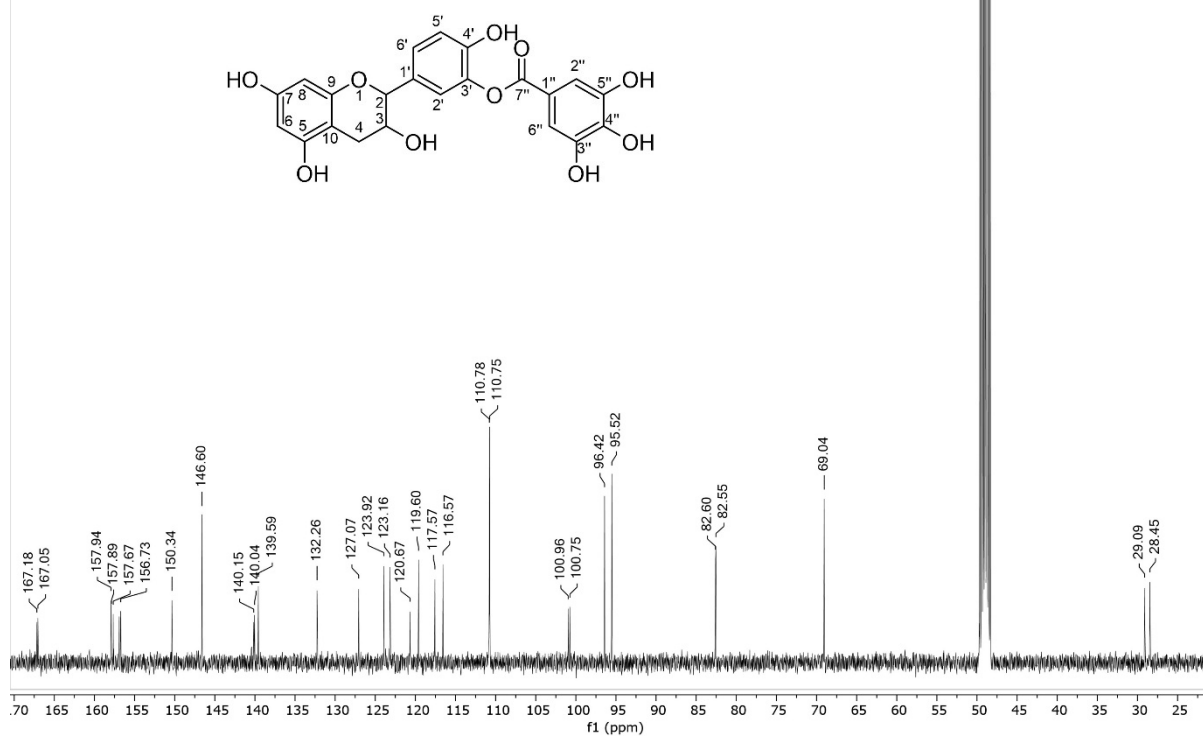

**Figure S6:** <sup>13</sup>C NMR spectrum of compound B21 purified by C18 HPLC from *K. wilmsii* twigs.

Supplement: Supplementary file 1 [file antibiotics-15-00141-s001.zip › Figure S6.pdf]

**B26**

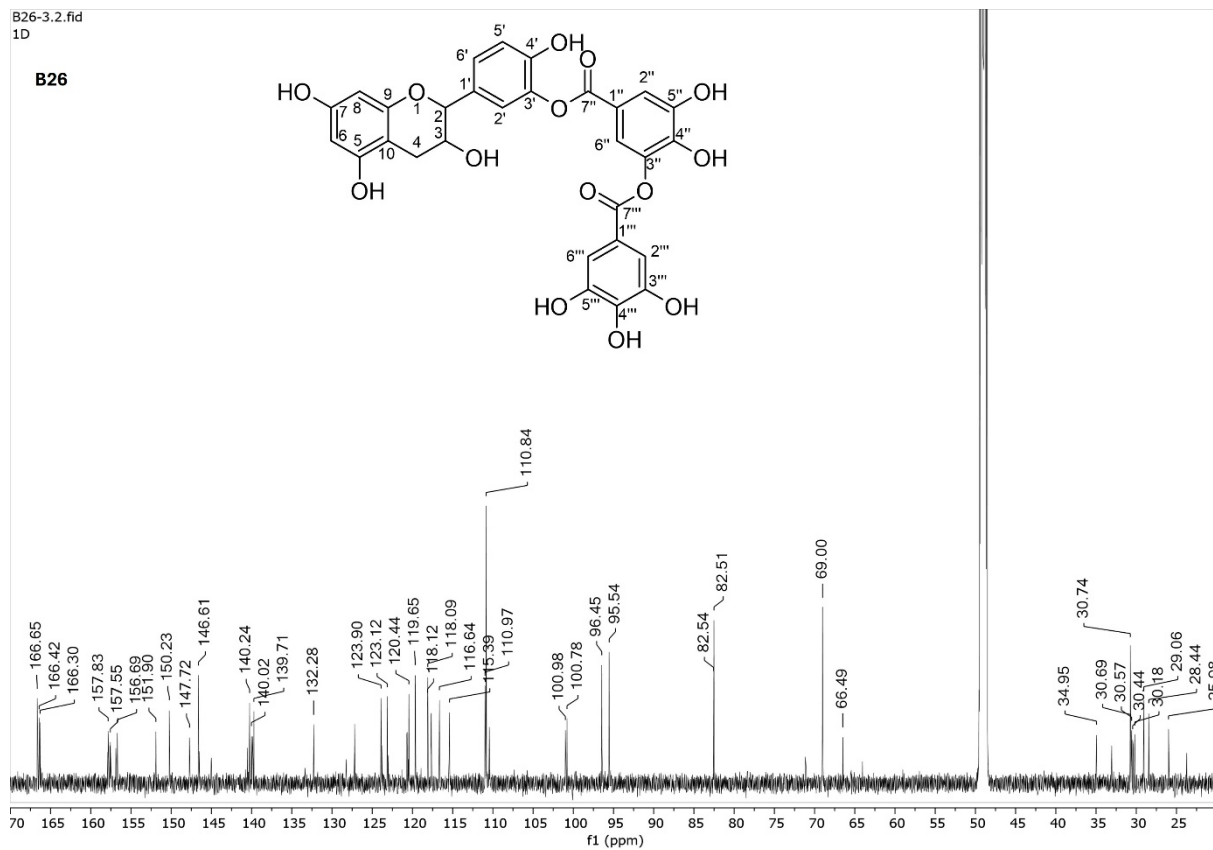

**Figure S7:** <sup>13</sup>C NMR spectrum of compound B26 purified by C18 HPLC from *K. wilmsii* twigs.

Supplement: Supplementary file 1 [file antibiotics-15-00141-s001.zip › Figure S7.pdf]

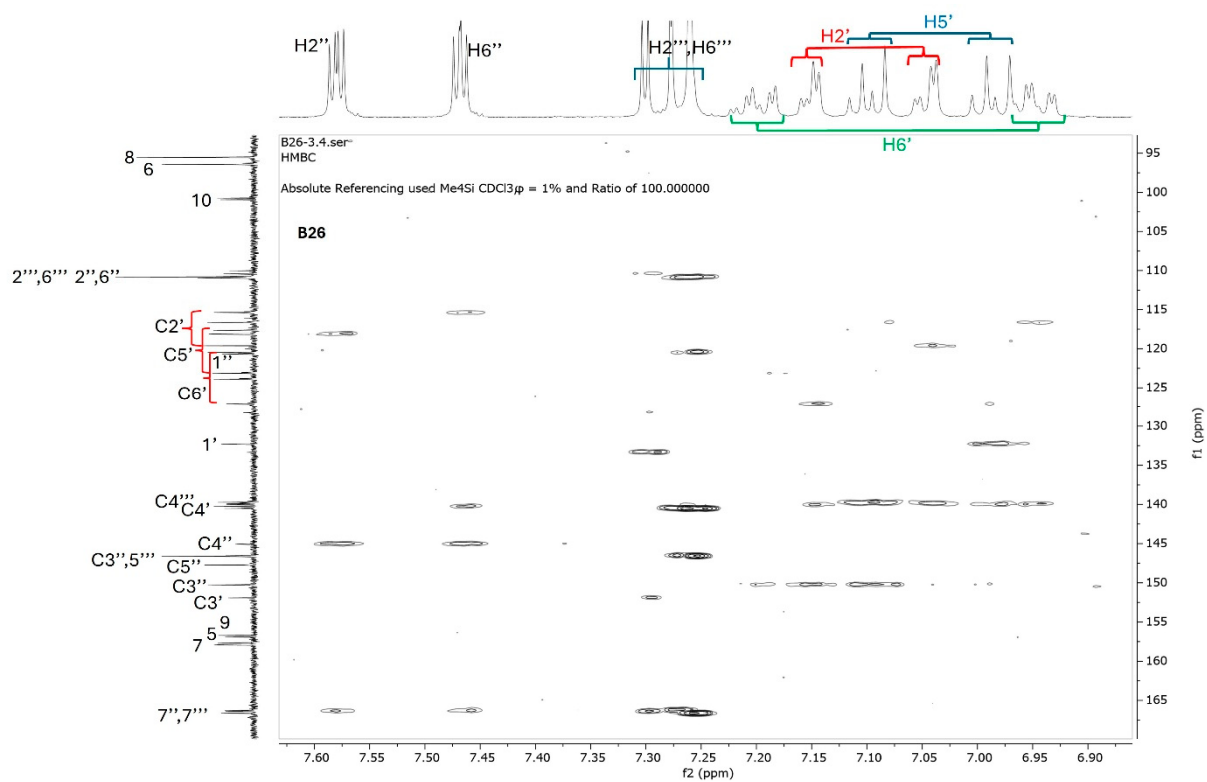

**Figure S9:** HMBC spectrum of compound B26 that was purified by C18 HPLC from *K. wilmsii* twigs.

Supplement: Supplementary file 1 [file antibiotics-15-00141-s001.zip › Figure S9.pdf]
